# MS-LTCAF: A Multi-Scale Lead-Temporal Co-Attention Framework for ECG Arrhythmia Detection

**DOI:** 10.3390/bioengineering12091007

**Published:** 2025-09-22

**Authors:** Na Feng, Chengwei Chen, Peng Du, Chengrong Gong, Jianming Pei, Dong Huang

**Affiliations:** 1 Department of Physiology and Pathophysiology, Air Force Medical University, No. 169 Changle West Road, Xi’an 710032, China; 2 Department of Biomedical Engineering, Air Force Medical University, No. 169 Changle West Road, Xi’an 710032, China; 3 Shaanxi Provincial Key Laboratory of Bioelectromagnetic Detection and Intelligent Perception, No. 169 Changle West Road, Xi’an 710032, China; 4 Innovation Research Institute, Xijing Hospital, Air Force Medical University, No. 169 Changle West Road, Xi’an 710032, China; 5 Department of Computer Fundamentals, Air Force Medical University, No. 169 Changle West Road, Xi’an 710032, China

**Keywords:** ECG arrhythmia detection, lead-temporal co-attention, multi-scale framework

## Abstract

Cardiovascular diseases are the leading cause of death worldwide, with arrhythmia being a prevalent and potentially fatal condition. The multi-lead electrocardiogram (ECG) is the primary tool for detecting arrhythmias. However, existing detection methods have shortcomings: they cannot dynamically integrate inter-lead correlations with multi-scale temporal changes in cardiac electrical activity. They also lack mechanisms to simultaneously focus on key leads and time segments, and thus fail to address multi-lead redundancy or capture comprehensive spatial-temporal relationships. To solve these problems, we propose a Multi-Scale Lead-Temporal Co-Attention Framework (MS-LTCAF). Our framework incorporates two key components: a Lead-Temporal Co-Attention Residual (LTCAR) module that dynamically weights the importance of leads and time segments, and a multi-scale branch structure that integrates features of cardiac electrical activity across different time periods. Together, these components enable the framework to automatically extract and integrate features within a single lead, between different leads, and across multiple time scales from ECG signals. Experimental results demonstrate that MS-LTCAF outperforms existing methods. On the PTB-XL dataset, it achieves an AUC of 0.927, approximately 1% higher than the current optimal baseline model (DNN_zhu’s 0.918). On the LUDB dataset, it ranks first in terms of AUC (0.942), accuracy (0.920), and F1-score (0.745). Furthermore, the framework can focus on key leads and time segments through the co-attention mechanism, while the multi-scale branches help capture both the details of local waveforms (such as QRS complexes) and the overall rhythm patterns (such as RR intervals).

## 1. Introduction

Cardiovascular diseases remain the leading cause of global mortality, accounting for approximately 19.8 million deaths annually. Among these, arrhythmia represents a common and potentially fatal cardiac manifestation [1,2]. Arrhythmia, defined as an abnormal heart rate or rhythm resulting from disturbances in cardiac electrical conduction, often serves as an early indicator of underlying cardiac pathologies and can precipitate severe complications such as heart failure, stroke, or sudden cardiac death if not diagnosed and managed in a timely manner [3].

In clinical practice, the electrocardiogram (ECG) is well-established as the gold standard for arrhythmia detection owing to its non-invasiveness, cost-effectiveness, and capacity to capture real-time cardiac electrical activity [4]. While single-lead ECGs offer essential rhythm information, multi-lead systems—particularly the standard 12-lead configuration—provide a spatially comprehensive perspective by recording electrical signals from multiple anatomical vantage points across the chest and limbs [5]. This multidimensional data captures regional heterogeneity in myocardial depolarization and repolarization, thereby facilitating more accurate localization of arrhythmic foci and improved discrimination among complex arrhythmia subtypes [6].

Early automated ECG diagnosis primarily relied on traditional machine learning methods such as Random Forest and Support Vector Machines (SVM) [7]. These approaches typically required manual feature engineering (e.g., RR interval, QRS complex width, ST segment slope), relied heavily on domain expertise, and involved cumbersome feature engineering processes with limited generalization capabilities. With the rapid advancement of deep learning, neural network-based ECG classification methods have achieved significant breakthroughs. In single-lead analysis, Liu et al. [8] demonstrated the feasibility of detecting arrhythmias using short-duration single-lead ECG via one-dimensional convolutional neural networks. Chen et al. [9] proposed reconstructing standard 12-lead ECGs from arbitrary single-lead ECGs to improve detection performance. However, this approach relies on statistical correlations rather than physiological spatial relationships, which may introduce reconstruction artifacts and limit clinical interpretability. Multi-lead analysis presents unique challenges, with effectively modeling spatial correlations between leads emerging as a critical issue. Some studies propose using 2D-CNN to treat multi-lead ECGs as image processing tasks, but this approach may fail to fully capture physiological relationships between leads [10]. In recent years, attention mechanisms have demonstrated significant potential in ECG analysis by enabling models to automatically focus on abnormal features in distinct regions, thereby improving classification accuracy. Taki Hasan Rafi [11] integrated attention modules into a convolutional architecture to enhance the representation of critical heartbeat segments. Elsheikhy et al. [12] added channel attention mechanisms to CNN to strengthen the representation of key diagnostic features in ECG signals while constructing lightweight models.

However, existing methods suffer from three major limitations: first, most studies focus on single-lead temporal features while neglecting spatial synergistic effects across multiple leads. Second, current attention mechanisms often process spatial or temporal dimensions independently, lacking effective coordination. Third, traditional models lack mechanisms to integrate multi-scale temporal features, limiting their ability to simultaneously capture local waveform details and global rhythmic patterns.

To address these challenges, we introduce the Multi-Scale Lead-Temporal Co-Attention Framework (MS-LTCAF). This framework is designed to effectively extract and analyze intra-lead and inter-lead features while leveraging a dual-attention mechanism to focus on the most relevant information and reduce redundancy. By integrating a Lead-Temporal Co-Attention Residual (LTCAR) module and the multi-scale branch structure, MS-LTCAF aims to enhance the performance of multi-lead ECG classification models.

Equipped with these perspectives, the contributions of this work are summarized as follows:

- The proposed MS-LTCAF provides a novel approach for end-to-end extraction and fusion of intra-lead, inter-lead, and multi-scale temporal features in multi-lead ECG classification.
- The LTCAR module, with its dual-path design and lead-time co-attention mechanism, enables the model to dynamically focus on key leads and time segments. The multi-scale branch structure effectively integrates short-, medium-, and long-range temporal features, achieving joint modeling of local waveform details and global rhythm patterns.
- This framework demonstrates excellent performance on the PTB-XL and LUDB datasets, offering a promising solution to improve the accuracy and efficiency of cardiovascular disease diagnosis.

## 2. Related Work

This section is divided into three parts: (1) an overview of the theoretical foundations of electrophysiology and multi-lead analysis; (2) a review of the application of deep learning and machine learning in arrhythmia detection; and (3) an exploration of the key role of attention mechanisms in improving classification accuracy in multi-lead analysis.

### 2.1. Theoretical Background of ECG Diagnosis

An electrocardiogram (ECG) represents the comprehensive vector manifestation of cardiac electrophysiological activity. Understanding its underlying principles forms the foundation for developing efficient, interpretable automated analysis algorithms. This section elucidates the core theoretical basis for effectively addressing this problem, tracing its origins from cardiac electrophysiology to modern deep learning methods, with particular emphasis on attention mechanisms.

#### 2.1.1. Physiological Basis of Electrocardiography

The rhythmic contractions of the heart are triggered by the depolarization and repolarization processes of cardiac muscle cells. The resulting bioelectric changes are conducted through bodily fluids to the skin surface, forming a waveform of potential differences that can be recorded by electrodes as an electrocardiogram (ECG). A standard cardiac cycle manifests on the ECG as a series of characteristic deflection waveforms, each corresponding to a specific phase of cardiac electrical impulse propagation:

- P wave: Reflects atrial depolarization. Abnormal morphology is often associated with atrial arrhythmias (e.g., atrial fibrillation) [13].
- QRS complex: Reflects rapid ventricular depolarization. Its width, morphology, and amplitude are critical for ventricular arrhythmias (e.g., premature ventricular contractions) and myocardial infarction [14].
- T wave: Reflects ventricular repolarization; abnormalities may indicate myocardial ischemia, electrolyte disturbances, etc. [15].
- PR interval, ST segment, and QT interval: These intervals and segments carry critical diagnostic information, reflecting atrioventricular conduction time, early ventricular repolarization, and total ventricular electrical activity duration, respectively.

Arrhythmias originate from disturbances in the initiation, propagation, or conduction velocity of cardiac electrical impulses. These abnormalities manifest as alterations in the morphology, duration, and rhythmic patterns of corresponding ECG waveforms, forming the physiological foundation for automated arrhythmia classification based on ECG analysis.

#### 2.1.2. The Value of Multi-Lead Electrocardiography and Spatial Information Theory

The standard 12-lead ECG records cardiac electrical activity from multiple angles (six axes in the frontal and horizontal planes), forming a spatial observation system. The placement of electrodes in different leads determines their sensitivity to electrical activity in specific cardiac regions, based on the principle of electrocardiographic vector projection:

- Inferior wall leads (II, III, aVF): Electrodes positioned on the lower limbs are particularly sensitive to detecting inferior wall myocardial infarction.
- Lateral wall leads (I, aVL, V5, V6): Critical for observing abnormalities in the lateral wall myocardium.
- Anterior interventricular leads (V1–V4): Advantageous for detecting ischemia or infarction in the anterior interventricular myocardium.
- aVR lead: Often provides unique information about the overall electrical axis of the heart, holding particular value in diagnosing widespread myocardial ischemia or arrhythmias.

Therefore, comprehensive analysis of multi-lead information is crucial for fully assessing cardiac electrical activity and accurately localizing anatomical lesions [16]. Compared to single-lead recordings that provide projections from only one spatial direction, multi-lead systems form a comprehensive spatial observation network. The multidimensional, complementary information they offer possesses theoretical advantages unmatched by single-lead systems in lesion localization and diagnostic redundancy. The core objective of this study is to achieve breakthroughs in classification performance by deeply mining this high-dimensional information system.

#### 2.1.3. The Theoretical Fit and Advantages of Attention Mechanisms

In ECG analysis tasks as described in Section 2.1.1 and Section 2.1.2, the diagnosis of abnormal heart rhythms (such as arrhythmias, atrial fibrillation, etc.) relies not only on temporal features like abnormal QRS complex morphology, P wave absence, or irregular occurrence, but is also closely related to spatial feature differences manifested across multiple leads. While traditional convolutional neural networks (CNNs) and recurrent neural networks (RNNs) possess automatic feature extraction capabilities, their inherent limitation lies in treating information across all temporal steps and spatial dimensions equally. This makes it difficult to explicitly capture feature structures with significant discriminative differences across different time periods and leads.

The introduction of attention mechanisms effectively addresses this shortcoming, with their computational principles aligning closely with the clinical logic of ECG diagnosis [17]. This mechanism dynamically calculates importance weights (Attention Weights) for each time point and lead within the input sequence in a data-driven manner, enabling the model to adaptively focus on local segments and spatial patterns most relevant to the diagnostic task. Explicit modeling of feature importance not only enhances classification accuracy but also improves model interpretability [18]. Visualizing attention weight distributions clearly identifies the key waveforms and lead regions underpinning model decisions, providing an intuitive clinical rationale for AI-assisted diagnoses and fostering physician trust in intelligent diagnostic systems. In this study, to further strengthen the model’s ability to jointly model spatiotemporal dependencies in multi-lead ECG signals, we introduce a synergistic architecture combining two attention mechanisms. This design aims to explicitly capture the interactive effects between temporal dynamics and spatial distributions, thereby representing multidimensional feature patterns under complex arrhythmias in a more comprehensive and discriminative manner.

### 2.2. Arrhythmia Detection Model

Current electrocardiogram (ECG) detection methods are mainly divided into two categories: machine learning and deep learning.

In the field of machine learning, Pan et al. [19] proposed a feature extraction scheme combining discrete wavelet transform, autocorrelation, and principal component analysis to extract seven features from ECG signals, employing a random forest algorithm for classification. Wang et al. [20] further integrated multiple features to achieve the mining and combination of local and global heartbeat characteristics, utilizing random forest, SVM, and BP neural networks for classification. To enhance feature representation, Fuadah et al. [21] decomposed ECG signals into multiple subbands using discrete wavelet transform (DWT), then extracted Hjorth descriptors and entropy-based features from each subband. They systematically compared the performance of five classifiers—kNN, SVM, random forest, ANN, and RBFN—under different parameter configurations. Luongo et al. [22] extracted optimal features from RR interval sequences in single-lead Holter ECGs using greedy forward selection techniques, achieving 73.5% accuracy with a decision tree classifier. Although these machine learning methods have demonstrated some effectiveness, their performance relies heavily on the quality of manually designed features. Manual features require extensive domain expertise and struggle to capture complex arrhythmia patterns and nonlinear feature relationships, limiting generalization in complex clinical scenarios.

With the advancement of deep learning technology, end-to-end automatic feature learning has achieved expert-level performance without requiring manual features [23,24,25]. Ezz [26] investigated the performance of five deep learning models—including Inception and DenseNet201—in single-lead ECG classification and diagnosis. Results showed that VGG16 paired with the V4 lead achieved 98.11% accuracy, demonstrating that single-lead ECG combined with deep learning can accurately diagnose cardiac diseases. Mathews et al. [27] explored unsupervised learning approaches, employing Restricted Boltzmann Machines (RBM) and Deep Belief Networks (DBN) to construct a deep learning framework for ECG signal classification. Kamyar et al. [28] proposed an innovative signal transformation method that converts single-lead ECG signals into graph structures via three approaches: natural visible graphs, horizontal visible graphs, and quantile graphs. They then utilized graph isomorphism networks for arrhythmia classification. Most of these studies focus on single-lead temporal feature analysis, neglecting spatial synergistic effects among multiple leads. Compared to single-lead ECGs, multi-lead ECGs contain richer cardiac activity information and can provide a more comprehensive assessment of cardiac abnormalities [29]. Bacevicius et al. [30] demonstrated through comparative studies that six-lead ECGs significantly outperform single-lead and PPG signals in detecting atrial fibrillation. However, multi-lead models exhibit non-orthogonal lead configurations, leading to redundant information from overlapping lead perspectives and partial loss of detailed features. Elisa et al. [31] proposed an entropy-based redundancy metric to reduce redundancy in 12-lead ECGs by removing highly redundant leads and applying linear transformations (e.g., PCA, IDT). Lai [32] employed a forward stepwise selection method to identify an optimal 4-lead subset comprising II, aVR, V1, and V4, effectively eliminating redundancy and enhancing model generalization. However, this approach cannot dynamically calibrate the importance of different leads or time segments. Furthermore, traditional models like CNN lack mechanisms to integrate multi-scale temporal features, limiting their ability to capture both local details and global patterns, thereby affecting diagnostic accuracy [33,34,35].

In summary, machine learning methods can achieve high accuracy but rely on features extracted from expert knowledge, making it difficult to capture complex rhythms. Deep learning methods can automatically extract key features and perform excellently in single-lead ECG diagnosis but often overlook synergistic multi-lead information, losing spatial features and limiting applicability in complex scenarios. Multi-lead ECGs offer richer information but also introduce redundancy and loss of detail. Existing methods to reduce redundancy cannot dynamically calibrate the importance of different leads or time segments, and traditional models like CNN cannot integrate multi-scale temporal features, making it difficult to balance local waveform details with global rhythm patterns, which affects diagnostic accuracy.

### 2.3. Attention Mechanism in Multi-Lead Electrocardiography

In recent years, attention mechanisms have been widely applied in ECG analysis, significantly enhancing the accuracy and interpretability of arrhythmia detection.

Regarding spatial attention, Huang et al. [36] proposed Guided Spatial Attention (GSA) and a CAM-based Spatial Guided Attention Mechanism (CGAM). By integrating clinical knowledge to create attention-guided labels for four ECG anomaly classification tasks, they significantly enhanced model classification performance and interpretability. Lu et al. [37] demonstrated the effectiveness of spatial attention in locating critical regions by predicting cardiac arrest in emergency departments using 12-lead ECG images, employing a spatial attention module and binary recall loss.

Regarding temporal attention, Manal [38] proposed a hybrid Transformer-CNN deep learning model. The self-attention mechanism within the Transformer architecture computes attention weights at each time step through a Query–Key–Value structure, enabling focus on temporal positions critical for anomaly detection. Ameet et al. [39] employed a self-attention autoencoder model to capture critical temporal dependencies in waveforms—such as QRS complexes and RR intervals—through self-attention mechanisms, primarily focusing on the temporal features of ECG signals.

Regarding channel attention, Marko [40] leveraged a channel attention mechanism to focus on key temporal features associated with atrial fibrillation in ECG and PPG data—such as patterns of RR interval irregularity over time—enabling efficient detection on wearable devices.

Such studies often focus on single-dimensional features when employing attention mechanisms to process electrocardiogram data. This isolated emphasis on either spatial or temporal characteristics struggles to comprehensively capture the interdependent relationships inherent in these signals. While this may enhance a model’s ability to identify abnormal cardiac activity patterns within specific dimensions, it risks limiting holistic interpretation by overlooking the interplay between these dimensions.

## 3. Methodology

The simultaneous extraction and analysis of intra-lead and inter-lead features can further improve the performance of multi-lead ECG diagnostic classification models. At the same time, the application of a dual-attention mechanism allows the model to focus on the most important information while ignoring less relevant features, thereby reducing information redundancy. To achieve this, we built a Multi-Scale Lead-Temporal Co-Attention Framework (MS-LTCAF). The signal is input into the MS-LTCAF, processed by Lead-Temporal Co-Attention Residual (LTCAR) Module and a multi-scale branch structure, and then sent to the classification head to output the results.

### 3.1. Detailed Description of the Multi-Scale Lead-Temporal Co-Attention Framework (MS-LTCAF)

The multi-lead ECG data X is entered into the proposed framework in the form of N × C × l × L tensors, where N denotes the number of samples, C represents the number of input channels, l indicates the number of leads (with a default value of 12), and L corresponds to the length of the signal sample. Following preprocessing, the signal undergoes feature extraction and classification through the network architecture. Ultimately, the classification result corresponding to the label Y is the result, with a dimensionality of N × K, where N is the number of samples and K is the total number of categories.

**Figure 1:**
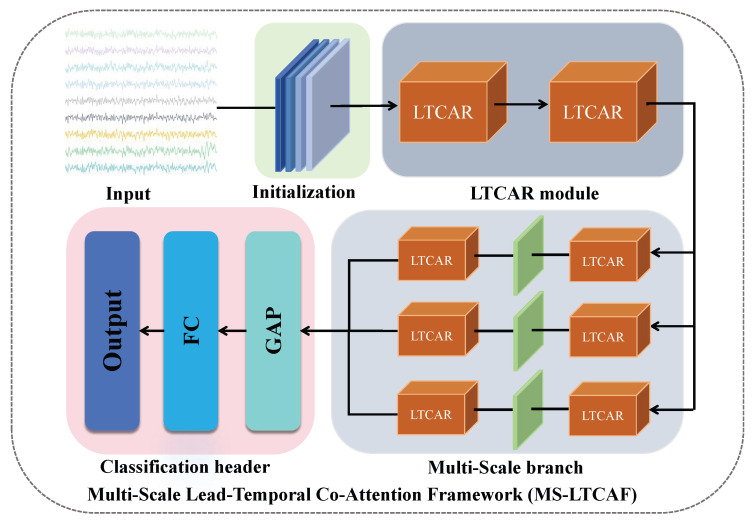
Multi-Scale Lead-Temporal Co-Attention Framework (MS-LTCAF). The ECG signal enters the initialization layer for preliminary feature extraction. It is then input into the multi-scale branch structure, which is composed of multiple LTCAR modules and convolutions for in-depth feature mining. Finally, the multi-scale features are concatenated and sent to the classification head for classification and prediction.

The MS-LTCAF consists of multiple LTCAR modules and a multi-scale branch structure. The multi-scale branch structure is composed of LTCAR modules with different parameters and 2D convolutions. The LTCAR module uses two convolution kernels of different sizes and an attention mechanism to extract intra-lead and inter-lead features. Skip connections are used to fuse features extracted from multiple paths efficiently. The general architecture of the framework is illustrated in Figure 1.

First, the signal is fed into the first initialization layer, which comprises the Convolution, BatchNorm, ReLU, and Dropout layers. The convolutional kernel of the initial convolution layer is (3, 15), which is capable of extracting both intra-lead and inter-lead features simultaneously. The BatchNorm layer performs batch normalization, which accelerates the training process and enhances the stability of the model. The introduction of the non-linear ReLU activation function enables the model to converge rapidly while preventing the vanishing gradient problem. However, to reduce the risk of overfitting, the Dropout layer is incorporated.

Subsequently, the output of the initialization layer flows into the first LTCAR module, each containing two paths. Path 1 is based on (k, n) convolutional kernels to compress along the center, and at the same time, lead attention is added to automatically learn the correlation between leads, and capture the synergistic effect of different lead combinations on specific pathological features. Path 2 is based on stacked m (1, n) convolutional kernels compressed along the time dimension, increasing the global channel dependence of temporal attention modeling, dynamically calibrating the importance of multi-channel features, and making up for the local limitation of convolution. At the same time, the model’s joint modeling ability of ECG local waveform and global rhythm is improved through jump connection.

The second LTCAR module, which has the same structure but a different number of channels, outputs directly to the multi-scale branch structure. This structure contains three parallel paths, each consisting of two LTCAR modules and a convolutional layer, using convolutional nuclei of different widths (n = 5, 7, 15) to capture ECG signatures at different time scales. This design enhances the model’s adaptability to different heart rates and waveform variations, thereby improving classification accuracy. Finally, the outputs of the three branches are concatenated and fed into global average pooling, which compresses the height and width of the feature map to 1 × 1. The resulting features are then passed through the final fully connected layers (Linear, BatchNorm, GELU, Dropout) for final classification prediction, outputting the score of each sample for each category.

### 3.2. Lead-Temporal Co-Attention Residual (LTCAR) Module

**Figure 2:**
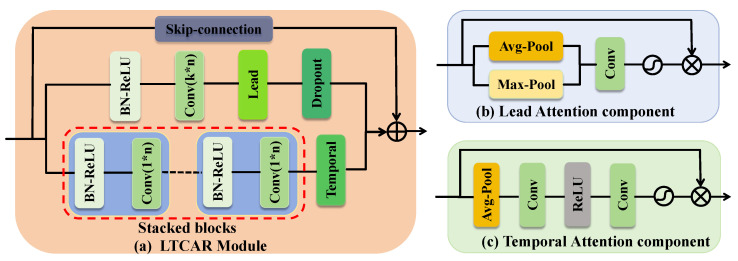
LTCAR Module. (**a**) LTCAR Module. (**b**) Temporal Attention component. (**c**) Lead Attention component.

To better extract intra-lead and inter-lead features and fully leverage available information, we propose the Lead-Temporal Co-Attention Residual (LTCAR) module. The LTCAR module can be flexibly added to any position in the network to improve the performance of the model. Its specific structure is shown in Figure 2.

Path 1 is the global–local feature path. After the input passes through the batch normalization and ReLU activation layers, a convolution kernel of size (k, n) is used to convolve the input, where k acts on the lead dimension and n acts on the time dimension to capture inter-lead correlations. Adding lead attention (as illustrated in Figure 2b and Equation (1)) generates spatial weights through max pooling and average pooling, enhances the response of key regions between leads, dynamically adjusts the feature importance of different positions, directs the model’s focus toward the most discriminative regions for the classification task, and models the collaborative variations among multiple leads. Finally, feature map Dropout is added to reduce the model’s dependence on specific signs, prevent overfitting, and improve its generalization ability.

(1) $$\begin{array}{cc} \; & F_{avg}\left(x\right)=\mathrm{AvgPool}\left(x\right)\\ \; & F_{1}=\mathrm{ReLU}\left(W_{1}\cdot F_{avg}\left(x\right)\right)\\ \; & F_{2}=\mathrm{Sigmoid}\left(W_{2}\cdot F_{1}\right)\\ \; & y=x⊗F_{2} \end{array}$$

Among them, *x* denotes the input feature map; $F_{\mathrm{avg}}\left(x\right)$ denotes the global average pooling operation; $W_{1}$ and $W_{2}$ denote the weight matrices of the two convolutional layers; r denotes the dimensionality reduction coefficient (reduction); ⊗ denotes the channel-wise multiplication operation; y denotes the output feature map after channel attention weighting.

Path 2 is the time-series feature deepening path. It stacks m (1, n) convolution kernels, focusing on the time dimension to extract local time-series features for a single lead. By stacking m convolutions, while reducing the number of parameters to accelerate the training process, multi-layer convolutions can extract more refined features layer by layer, capture fine-grained time changes, and form a richer time-series feature space, which is helpful for identifying subtle features in ECG signals. Introducing temporal attention (as shown in Figure 2c and Equation (2)) can generate channel weights through global average pooling, identify channels that extract key time patterns, suppress noise and redundant information, strengthen key channels, and finally form fine-grained temporal feature modeling.

(2) $$\begin{array}{cc} \; & F_{max}\left(x\right)=\mathrm{MaxPool}\left(x,\dim=1\right)\\ \; & F_{avg}\left(x\right)=\mathrm{MeanPool}\left(x,\dim=1\right)\\ \; & F_{cat}=\mathrm{Concat}\left(F_{max}\left(x\right),F_{avg}\left(x\right),\dim=1\right)\\ \; & F_{attn}=\mathrm{Sigmoid}\left(W\cdot F_{cat}\right)\\ \; & y=x⊗F_{attn} \end{array}$$

Among them, x denotes the input feature map; $F_{\max}\left(x\right)$ represents the maximum pooling operation along the channel dimension; $F_{\mathrm{avg}}\left(x\right)$ represents the average pooling operation along the channel dimension; $F_{\mathrm{cat}}$ denotes the concatenation operation along the channel dimension; W represents the weight matrix of the convolutional layer (using a 7 × 7 convolution kernel); ⊗ indicates the element-wise multiplication operation; and y represents the output feature map after spatial attention weighting.

Skip connections allow gradients to flow directly back to earlier layers, thereby alleviating the problem of vanishing gradients. They can also add the input feature map to the feature map after multi-layer processing, preserving the original information while promoting feature flow. The dual-path architecture, combined with the dual-attention collaborative mechanism, more effectively exploits the relationships between intra-lead and inter-lead ECG features, thereby optimizing the model’s classification performance.

### 3.3. Multi-Scale Branch Structure

ECG signals exhibit features across multiple time scales, ranging from rapidly changing QRS complexes to relatively slow P waves and T waves. Single-scale branch convolution kernels cannot comprehensively capture these features. Therefore, we propose a multi-scale branch structure that can simultaneously capture rhythmic features at different time scales in ECG signals, enabling the model to concurrently learn local details and global rhythmic patterns, thereby enhancing the discriminative ability for different arrhythmia categories.

Each branch comprises two LTCAR modules and one convolutional layer. In each branch, the first LTCAR module adopts a larger convolution kernel (3, n), where k = 3 models the dependencies of local lead combinations in the spatial dimension (inter-lead), while n = 5, 7, 15 covers ECG waveform features across different ranges in the temporal dimension, thereby extracting extensive spatiotemporal contextual information. The intermediate convolutional layer strengthens feature representation, prevents multi-branch information during transmission, and improves feature robustness. The second LTCAR module employs a smaller convolution kernel (1, n), where k = 1 only focuses on feature changes within a single lead to avoid inter-lead interference, while n = 5/7/15 maintains multi-scale temporal modeling. The smaller convolution kernel refines local feature extraction, compensating for the potential blurring introduced by large convolution kernels, and more accurately captures detailed features such as steep QRS transitions and minor ST-segment shifts.

The three branches employ convolution kernels of different sizes, specifically (3 × 5, 1 × 5), (3 × 7, 1 × 7), and (3 × 15, 1 × 15). By setting k = 3 and k = 1, multi-level spatial modeling is achieved, ranging from capturing correlations among local lead combinations to focusing on lead-specific dependencies of individual leads. This enhances the capability to detect inter-lead dependencies and local abnormal changes. In the temporal dimension, windows of n = 5, 7, and 15 cover short-, medium-, and long-range temporal features, thus capturing various types of temporal dependencies. This design allows simultaneous attention to local details (e.g., individual heartbeat morphology) and global rhythms (e.g., RR-interval variations), thereby improving adaptability to different heart rates and waveform variations.

## 4. Experiments

In this section, the performance of the proposed model is comprehensively analyzed and evaluated. Two public datasets—PTB-XL and the Lobachevsky University Electrocardiography database (LUDB)—are used to verify the classification effect of the model on multi-lead ECG data. The model is also compared with existing methods. In order to verify the effectiveness of the proposed model components and mechanisms, ablation experiments are conducted to highlight the contribution and importance of each component, which can further optimize the model structure and improve the overall performance.

### 4.1. Data

Data 1: PTB-XL ECG dataset

The PTB-XL ECG dataset [41] contains 21,837 clinical 12-lead ECGs from 18,885 patients. Each recording is 10 s in length, with sample frequencies of 500 Hz and 100 Hz and 16-bit resolution. The dataset was 52% male and 48% female, with ages ranging from 0 to 95 years (median 62 years; interquartile range: 22). The dataset was derived from a comprehensive collection of many different concurrent pathologies and also from mostly healthy control samples. Raw waveform data were annotated by up to two cardiologists who assigned multiple possible ECG statements to each record. In total, 71 different ECG statements met the SCP-ECG criteria, covering 44 diagnostic, 19 formal and 12 rhythmic statements, with four statements belonging to both diagnostic and formal categories. This study mainly focuses on the use of diagnostic statements to determine normal and abnormal ECG signals, and the official dataset aggregates 44 diagnostic statements into 5 superclass diagnostic labels (NORM, MI, STTC, CD, HYP) because each record may have multiple labels, and the sum of the labels exceeds the number of records; however, the original dataset contains samples without a clear diagnostic category, and in order to ensure data consistency and comparability, this study improves the data quality and training effect by filtering invalid samples, The final dataset label distribution used in this study is shown in Table 1.

The PTB-XL dataset was obtained by stratified sampling while respecting patient assignment, i.e., all records for a particular patient were assigned the same first fold. The records in the 9th and 10th fold have been evaluated manually at least once and have higher label quality. Therefore, this study adopted the official recommendation of using folds 1–8 as the training set, fold 9 as the validation set, fold 10 as the test set, and selects data with a sample frequency of 100 Hz.

Preprocessing mainly includes signal truncation (unified to a 10-s length) and Robust Scaling normalization based on the median and interquartile range (IQR). During training, data augmentation methods such as random time shifting, scaling, and slight noise are applied in real time to enhance the generalization ability of the model. This process relies on the well-established wfdb library and NumPy for implementation. The computational load mainly comes from one-time data loading (disk I/O), while the memory computation overhead for tasks like normalization is extremely low.

Data 2: Lobachevsky University Electrocardiography database (LUDB)

The Lobachevsky University Electrocardiography Database (LUDB) [42] is an ECG signal database with a sampling frequency of 500 Hz. It contains 200 records of 10-s 12-lead ECG signals, including 85 females and 115 males, with ages ranging from 11 years to over 89 years (average: 52 years). Cardiologists manually annotated each lead in each data record, marking the boundary and peak of P waves, T waves, and QRS complexes. This dataset can be classified in various ways. In this study, the specified heart rate types are used to determine whether the ECG signals are normal or abnormal.

Meanwhile, to ensure data quality and training effectiveness, this study adopts an automatic QRS detection process based on the improved Pan–Tompkins algorithm. It considers a time interval of 0.4 s before and 0.6 s after the peak of a given QRS complex to extract complete cardiac cycles. This approach eliminates the need for labor-intensive and subjective manual heartbeat annotation, thus ensuring the reproducibility and efficiency of data construction. After filtering out invalid samples, QRS region-specific augmentation (e.g., adding noise only in the QRS region), lead-specific gain, and time warping were introduced as data augmentation methods. The final label distribution is provided in Table 2. This study adopts five-fold cross-validation.

### 4.2. Implementation Details

During data preprocessing, Robust Scaling is applied for normalization to suppress interference from outliers, such as transient interruptions in specific leads. Data augmentation is performed in real time throughout the training process. For parameter initialization, the He method is adopted to stabilize input distribution for the ReLU activation function and accelerate convergence. During training, the AdamW optimizer and Focal Loss are used in combination to address class imbalance and hard example recognition; this is paired with a cosine annealing learning rate scheduler to ensure the model escapes local optima. Regularization is implemented via Dropout (with a probability of 0.2–0.4) and gradient clipping (with a threshold of 10.0) to enhance the model’s generalization ability. All experiments are conducted on a server equipped with an NVIDIA Tesla V100 PCIe 32 GB GPU and implemented based on the PyTorch 2.50 framework.

### 4.3. Evaluation Indicators

For multi-label classification problems, evaluation metrics need to simultaneously consider the prediction of multiple labels for each sample. There are sample-based metrics and label-based metrics. In this study, label-based metrics are adopted. Model performance is evaluated using the Area Under the Curve (AUC), Accuracy, F1-score, Precision, and Recall. The calculation formulas for these metrics are as follows:

(3) $$\begin{array}{c} \mathrm{Accuracy}=\frac{TP+TN}{TP+TN+FP+FN} \end{array}$$

(4) $$\begin{array}{c} F1=2\times \frac{\mathrm{Precision}\times \mathrm{Recall}}{\mathrm{Precision}+\mathrm{Recall}} \end{array}$$

(5) $$\begin{array}{c} \mathrm{Precision}=\frac{TP}{TP+FP} \end{array}$$

(6) $$\begin{array}{c} \mathrm{Recall}=\frac{TP}{TP+FN} \end{array}$$

Among them, AUC refers to the area under the receiver operating characteristic (ROC) curve. TP, FP, TN, and FN represent true positive, false positive, true negative, and false negative, respectively.

### 4.4. Comparative Translation with Other Baseline Methods

#### 4.4.1. Baseline

A wide variety of arrhythmia detection models have been proposed. To verify the performance of the model presented in this study, we compare it with several existing models with better performance.

ResNet34 (He et al., 2016) [43]: ResNet34 has achieved good results across multiple classification tasks and is widely used as a basic framework. In this study, it is reproduced as 1D and 2D arrhythmia detection models.

DNN_zhu (Zhu et al., 2020) [44]: The DNN_zhu model performs excellently in single-label and multi-label ECG diagnosis, with most indicators exceeding the average performance of human physicians.

DSE-ResNet (Li et al., 2022) [10]: DSE-ResNet has certain advantages in detecting some arrhythmias using multi-lead 2D-ECG.

SE-ResNet (Park et al., 2022) [45]: SE-ResNet has been applied to single-lead ECG classification, with particularly high accuracy reported on Lead II data. In this study, we reproduce both as a single-lead model using only Lead II and a model using 12 leads.

LightX3ECG (Le et al., 2023) [46]: This model is specifically designed to identify various cardiovascular abnormalities through three-lead ECG data (I, II, and V1 leads).

#### 4.4.2. Results

**Figure 3:**
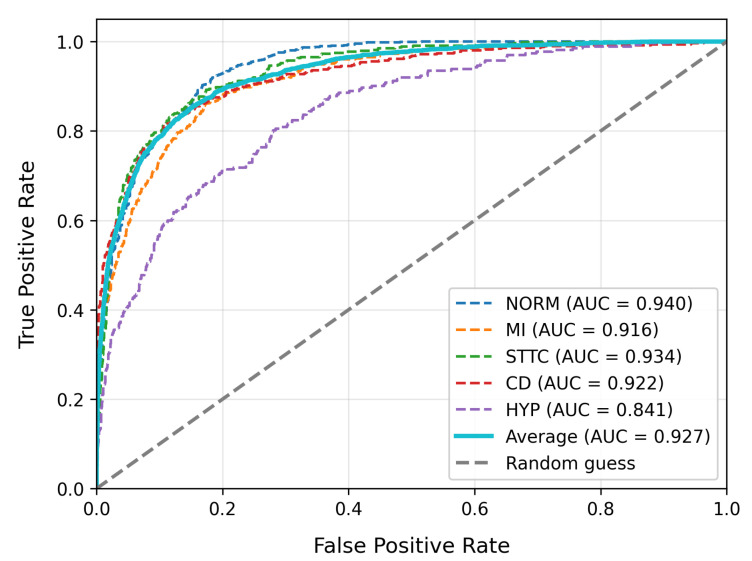
The ROC curve of MS-LTCAF on PTB-XL dataset.

**Figure 4:**
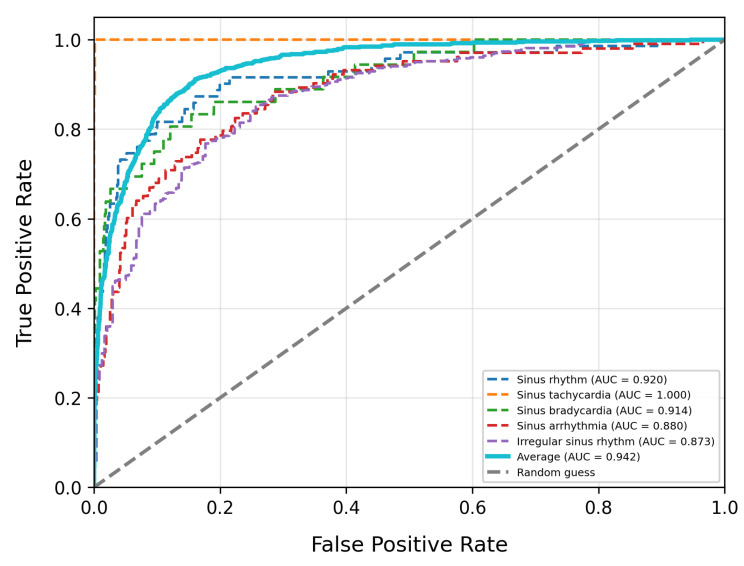
The ROC curve of MS-LTCAF on LUDB dataset.

To evaluate model performance, we compare our approach with existing high-performing baseline models. Table 3 summarizes the overall performance, model complexity, and inference performance tests of our model alongside other baseline models on the PTB-XL dataset. Table 4 provides the detailed classification report for our model on PTB-XL, and Table 5 displays the results on the LUDB dataset. The ROC curves for our model on PTB-XL and LUDB are shown in Figure 3 and Figure 4, respectively.

In the evaluation on the PTB-XL dataset, as shown in Table 3, the MS-LTCAF demonstrates significant advantages. It achieves an AUC of 0.927, which is significantly better than ResNet34_1d (0.908), ResNet34_2d (0.911), and DNN_zhu (0.918) (*p* < 0.05). Its accuracy (0.877) is comparable to that of the optimal baseline (ResNet34_1d 0.882) (*p* > 0.1). Furthermore, its F1-score (0.745) is significantly higher than those of the baseline models (*p* < 0.05), indicating that MS-LTCAF achieves an optimal balance between precision and recall while maintaining classification stability.

From the perspective of computational complexity and resource efficiency, MS-LTCAF achieves a favorable balance between parameter size and memory usage. With 24.585 million parameters, it exceeds lightweight models such as DNN_zhu (3.681 million) but remains considerably smaller than the SE-ResNet family (approximately 58 million). This moderate parameterization enhances its representational capacity, leading to superior performance on key metrics—including an AUC of 0.927, an F1-score of 0.745, and a precision of 0.806—ranking it among the top-performing models in discriminative power and classification robustness. In terms of memory consumption, MS-LTCAF requires only 0.22 GB, comparable to the SE-ResNet series (0.26 GB) and significantly lower than DSE-ResNet (0.48 GB). Although some models, like LightX3ECG (0.03 GB), consume less memory, they underperform MS-LTCAF across multiple evaluation dimensions. Thus, MS-LTCAF strikes an effective trade-off, using modest increases in parameters and memory to deliver substantial gains in overall classification accuracy. This makes it particularly suitable for practical applications where both high performance and resource efficiency are required.

Table 4 presents specific ECG type evaluation metrics on the PTB_XL dataset. The model demonstrates satisfactory classification performance across most categories, with the highest F1-score (0.84) achieved for the Norm type, accompanied by precision and recall rates of 0.83 and 0.86, respectively. This indicates strong capability in identifying normal ECGs. However, metrics for the HYP category are notably lower (F1 = 0.39, Recall = 0.27). This discrepancy may stem from the fact that ECG alterations associated with hypertension often overlap with other clinical conditions, resulting in higher feature complexity that impairs the classifier’s discriminative power.

In the LUDB dataset evaluation, MS-LTCAF demonstrates outstanding performance. The model achieves an AUC of 0.942, showing stronger discriminative power than baseline models such as ResNet34_1d (0.870) in distinguishing positive from negative ECG samples, and more accurately capturing signal differences. Its accuracy reaches 0.920, outperforming SE-ResNet12 (0.903), which indicates lower error rates and higher stability when classifying overall ECG data. The F1-score is 0.745, with precision at 0.794 and recall at 0.701. All three metrics outperform most baselines, collectively demonstrating its outstanding classification performance and robustness in ECG signal analysis tasks.

From the perspective of computational complexity and resource efficiency, MS-LTCAF achieves an efficient design in terms of parameter count (24.585M) and memory consumption (0.16 GB). Its parameter count is significantly lower than that of the SE-ResNet series (58.147M), and its memory consumption is only about 1/7th (1.18 GB), demonstrating extremely high memory efficiency. Although its parameter count exceeds that of lightweight models such as DNN_zhu (3.682M) and LightX3ECG (5.583M), MS-LTCAF substantially outperforms these models across all key performance metrics. This highlights its ability to deliver significant performance gains with only moderate parameter growth. Such efficiency makes MS-LTCAF particularly well suited for deployment in resource-constrained practical scenarios, providing an effective solution for achieving high-precision ECG signal classification.

### 4.5. Ablation Study

To verify the effectiveness of the proposed model (Lead-Temporal Co-Attention (LTCA) mechanism, multi-scale branch structure, and LTCAR module), ablation experiments were conducted. By modifying key structures, we evaluated the impact of each component on classification performance and quantified its contribution to the overall model behavior.

#### 4.5.1. Effectiveness of Lead-Temporal Co-Attention Mechanism

**Figure 5:**
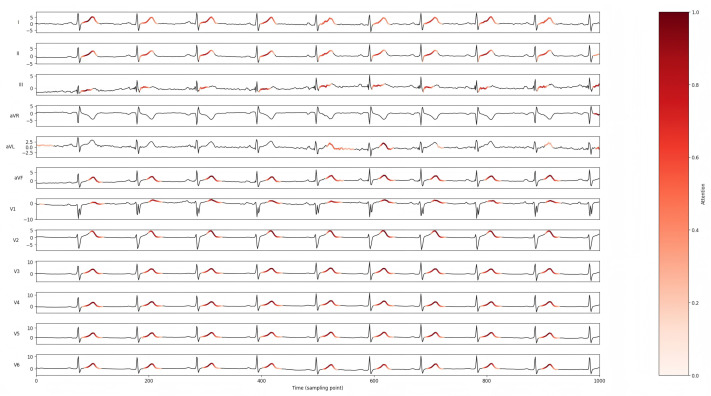
The Grad-CAM of MS-LTCAF on a NORM sample.

**Figure 6:**
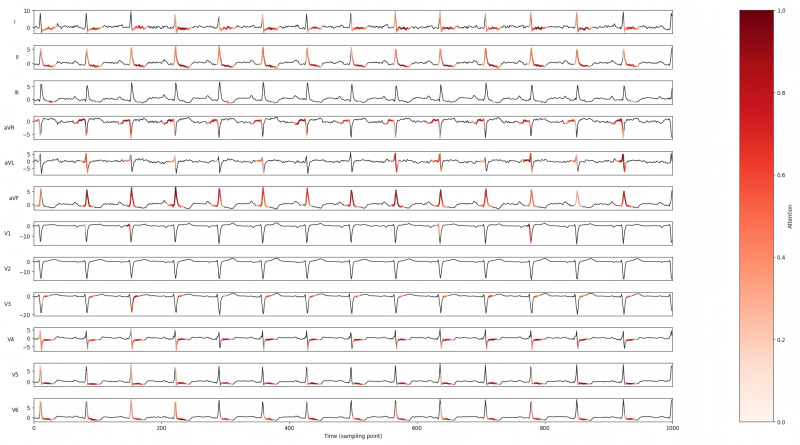
The Grad-CAM of MS-LTCAF on a STTC sample.

To verify the effectiveness of the LCTA Mechanism, we adopted the same training strategy and parameter configuration to differentiate only the attention module. In this experiment, the influence of attention mechanism on the overall performance of the model was explored by constructing a single attention variant (which contained only temporal or lead attention) and a non-attention variant. Their results were compared against the original model incorporating the LTCA mechanism. Furthermore, Grad-CAM visualization were employed to further visualize the effectiveness of attention. The results are shown in Table 6, and Figure 5 and Figure 6.

The experimental findings confirm the synergistic effect of combining lead attention and temporal attention, which can effectively enhance the model’s ability to extract and discriminate complex features, and then significantly improve the classification performance. Furthermore, through the visualization results, the role of the attention mechanism can be further verified, and the focused feature areas between and within different leads can be visually displayed.

#### 4.5.2. Effectiveness of the LTCAR Module

**Figure 7:**
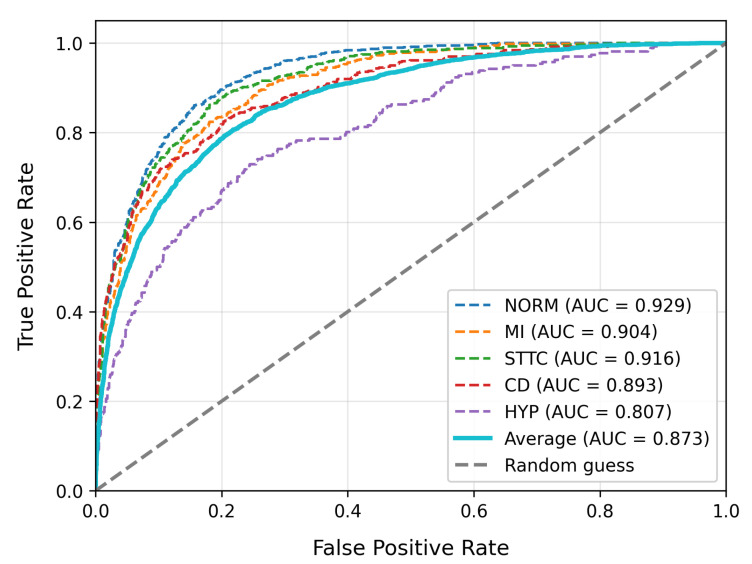
The ROC curves of the ablation experiment of the convolutional operation.

**Figure 8:**
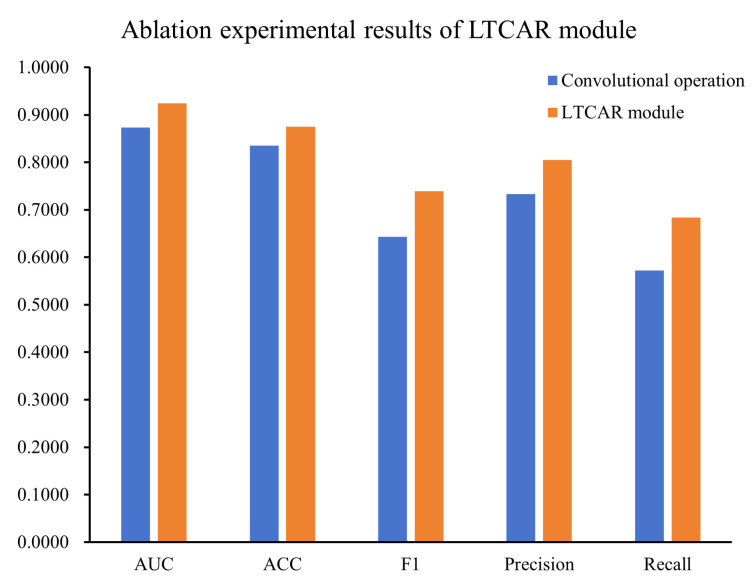
Comparison results of convolutional operation and LTCAR module in the ablation experiment.

In classification tasks involving spatiotemporal features such as ECG, the LTCAR module used in our framework contributes to improving model performance through dual-path convolution. To evaluate the effectiveness of the LTCAR module, we replaced it with a convolutional operation while keeping the overall architecture of the model, training strategy, and hyperparameter configuration completely consistent to ensure the rigor of the experiment. The experimental results are shown in Figure 7 and Figure 8.

The experimental results show that the LTCAR module exhibits significant advantages over the traditional convolutional operation in ECG classification tasks. The traditional convolutional operation relies on fixed-size convolution kernels for feature extraction, resulting in insufficient completeness of feature extraction and limited modeling capabilities. In contrast, the dual-path structure of the LTCAR module can simultaneously perform collaborative modeling from two dimensions: the dynamic evolution of time series and the local correlation of spatial features, achieving more comprehensive information capture. Experiments have confirmed the effectiveness of the LTCAR module in ECG classification tasks.

#### 4.5.3. Effectiveness of Multi-Scale Branch Structure

**Figure 9:**
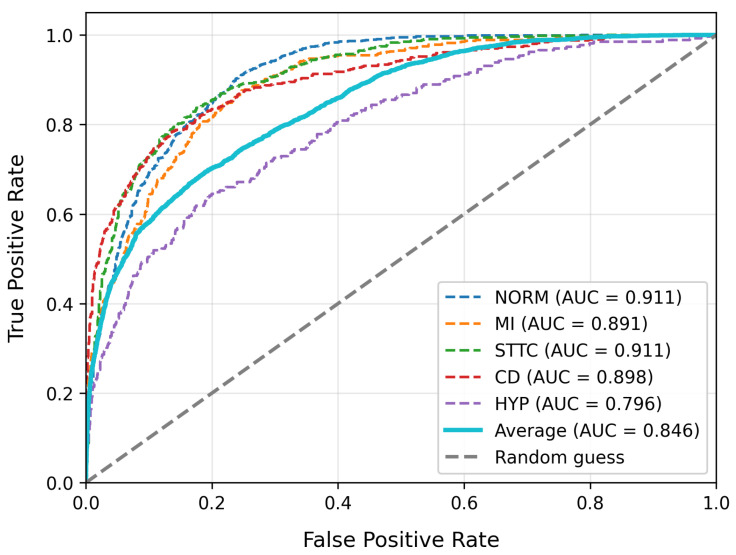
ROC curves of the ablation experiment of the single-scale branch structure.

**Figure 10:**
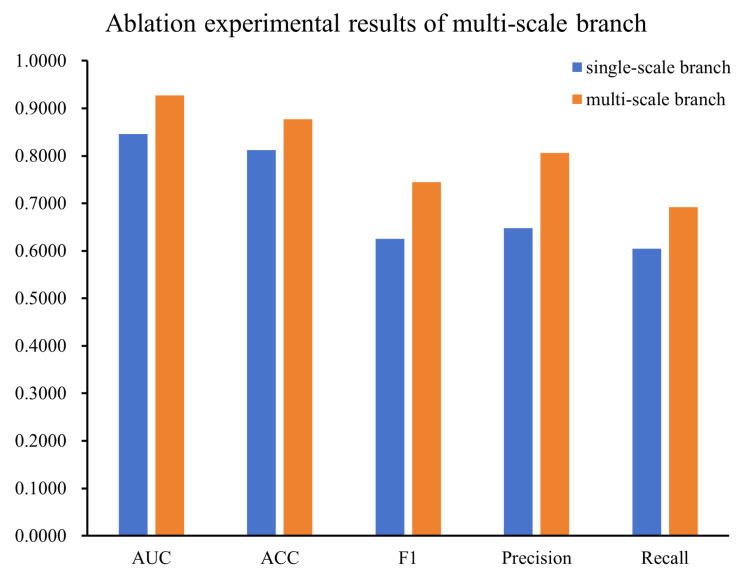
Comparison results of single-scale branch and multi-scale branch structure in the ablation experiment.

To evaluate the necessity of the multi-scale branch structure, we conducted experiments by comparing the performance differences between the original multi-branch structure and the simplified single-branch structure. In this experiment, to eliminate interference factors caused by extreme scales, only the intermediate-scale branch (branch2, n = 7) was retained, and the other two branches were removed. This ensured that the comparative analysis was conducted under the control of a single variable. The experimental results are shown in Figure 9 and Figure 10.

The experimental results indicate that the multi-scale branch structure not only effectively avoids the scale bias problem that may be caused by a single branch but also gives full play to the complementary advantages of features at different time scales. This structure is irreplaceable in ECG tasks and an indispensable key element for improving model performance.

## 5. Discussion

The MS-LTCAF proposed in this study effectively addresses issues such as inter-lead information redundancy, incomplete capture of temporal features, and insufficient local–global modeling in multi-lead ECG classification. By integrating the Lead-Temporal Co-Attention (LTCAR) module and the multi-scale branch structure, the model achieves excellent performance on both the PTB-XL and LUDB datasets. Compared with existing baseline models, MS-LTCAF achieves an AUC of 0.924, an F1-score of 0.740, and a precision of 0.805, verifying its advancement in multi-lead ECG classification tasks.

The core innovation of MS-LTCAF lies in the integration of the LTCAR module and the multi-scale branch structure. The LTCAR module models inter-lead correlations and intra-lead temporal features, respectively, through a dual-path design: Path 1 utilizes lead attention to dynamically enhance the synergistic effect of key lead combinations, while Path 2 focuses on important time segments through temporal attention. Together, these mechanisms reduce noise interference, highlight pathological features, and overcome the limitations of traditional models (such as SE-ResNet and ResNet34) that focus on a single lead or fixed time window. This design improves sensitivity to subtle features, such as ST-segment changes in myocardial infarction (MI) and waveform abnormalities in conduction disturbances (CD). Moreover, the ablation experiment shows that by replacing the (LTCAR) module with a traditional convolutional operation, the model’s recognition accuracy for conduction disturbance (CD) decreases (see Figure 7 and Figure 8 for details). This indicates that the convolutional operation limits its representation of ECG signals to a single dimension, making it difficult to fully capture complex waveform changes and spatial distribution patterns at different time scales. This underscores the advantage of the dual-path (LTCAR) module in modeling spatiotemporal correlations.

The multi-scale branch structure covers short-, medium-, and long-range temporal features through convolution kernels of different sizes (ranging from 3 × 5, 1 × 5 to 1 × 15), focusing on the micro-details, intermediate-level features, and macro-trends of ECG signals, respectively. This enables the model to extract local waveform details (e.g., QRS complexes) while simultaneously recognizing global rhythm patterns (e.g., sinus rhythm). In contrast, a single-scale branch structure struggles to simultaneously accommodate feature information at different levels, leading to the omission of key signal features. Ablation results (see Figure 9 and Figure 10 for details) show that reducing the architecture to a single-branch structure degrades performance, highlighting the indispensability of the multi-scale approach.

Notably, our MS-LTCAF model achieved the optimal performance on the LUDB dataset, while its advantage was relatively modest on the PTB-XL dataset (though its performance still remained comparable to state-of-the-art methods). We attribute this phenomenon to two key differences between PTB-XL and LUDB: one lies in task complexity and label granularity, and the other in dataset scale and model capacity. In terms of task complexity and label granularity, PTB-XL is a multi-label diagnostic dataset, where many records simultaneously contain multiple arrhythmias. This requires models to learn highly complex and overlapping electrophysiological patterns, relying heavily on comprehensive judgments of global context and subtle waveform variations. In contrast, LUDB is essentially a morphological classification dataset, focusing on distinguishing mutually exclusive rhythm categories with distinct morphological differences. Regarding dataset scale and model capacity, PTB-XL (with approximately 27,826 records) is substantially larger than LUDB (with around 3869 records). The massive volume of data in PTB-XL enables large baseline models to learn complex coexisting patterns, which offsets the advantages of the innovative architecture to a certain extent. Conversely, on the smaller-scale LUDB dataset, the superior data efficiency and feature-focusing capability of MS-LTCAF—realized through the collaborative attention mechanism—are fully exerted, allowing it to outperform baseline models that rely on extensive data fitting by a significant margin.

Although this study provides a new method for arrhythmia detection, certain limitations remain. The model’s recognition performance for rare categories (such as abnormal heart rhythms in LUDB) leaves room for improvement and needs further optimization through data augmentation or transfer learning. These samples are severely underrepresented in the dataset, which may lead to misclassifications due to insufficient training, with the classification performance not evaluated independently. Furthermore, although the leads and time regions focused on by the model were visualized through Grad-CAM, the decision logic of the attention mechanism was not analyzed in detail, lacking more fine-grained interpretability.

In summary, MS-LTCAF provides a new paradigm for multi-lead ECG classification. Its Lead-Temporal Co-Attention (LTCAR) module and multi-scale branch structure lay a technical foundation for the automated diagnosis of cardiovascular diseases, which is expected to play an important role in clinical auxiliary diagnosis.

## 6. Conclusions

This study proposes a Multi-Scale Lead-Temporal Co-Attention Framework (MS-LTCAF) for multi-lead ECG classification. The framework achieves end-to-end extraction and fusion of intra-lead, inter-lead, and multi-scale features through the Lead-Temporal Co-Attention (LTCAR) module and multi-scale branch structure, effectively addressing inter-lead information redundancy, incomplete temporal feature capture, and insufficient local–global modeling. Through the dual-path LTCAR design and the Lead-Temporal Co-Attention Mechanism, the model dynamically focuses on key leads and time fragments. The multi-scale branch structure integrates short-, medium- and long-range time features to achieve joint modeling of local waveform details and global rhythm patterns. The excellent performance on the PTB-XL and LUDB datasets demonstrates that MS-LTCAF provides an efficient solution for multi-lead ECG classification, with the potential to improve the accuracy and efficiency of cardiovascular disease diagnosis and serve as a valuable clinical aid.

## Figures and Tables

**Table 1:** Label distribution of PTB-XL superclass dataset.

Type	Description	Number
NORM	Normal ECG	9514 (9528)
MI	Myocardial Infarction	5469 (5486)
STTC	ST/T Change	5235 (5250)
CD	Conduction Disturbance	4898 (4907)
HYP	Hypertrophy	2649 (2655)

**Table 2:** Label distribution of LUDB dataset.

Rhythms	Number of ECGs	Samples
Sinus rhythm	143	2675
Sinus tachycardia	4	97
Sinus bradycardia	25	517
Sinus arrhythmia	8	183
Irregular sinus rhythm	2	43
Abnormal rhythm	19	354

**Table 3:** Model evaluation of the PTB-XL dataset.

Method	AUC	Accuracy	F1	Precision	Recall	Params	Memory
ResNet34_1d [43]	0.908	0.882	0.726	0.778	0.691	16.604M	0.1 GB
ResNet34_2d [43]	0.911	0.879	0.714	0.722	0.706	21.281M	0.11 GB
DNN_zhu [44]	0.918	0.890	0.762	0.758	0.774	3.681M	0.03 GB
DSE-ResNet [10]	0.827	0.784	0.626	0.568	0.697	3.404M	0.48 GB
SE-ResNet1 [45]	0.889	0.862	0.653	0.753	0.599	58.145M	0.26 GB
SE-ResNet12 [45]	0.923	0.880	0.725	0.772	0.694	58.150M	0.26 GB
LightX3ECG [46]	0.920	0.884	0.719	0.795	0.681	5.447M	0.03 GB
MS-LTCAF (Ours)	0.927	0.877	0.745	0.806	0.692	24.585M	0.22 GB

**Table 4:** Classification report of the PTB-XL dataset.

Type	F1	Precision	Recall
Norm	0.84	0.83	0.86
MI	0.68	0.81	0.58
STTC	0.75	0.81	0.71
CD	0.74	0.79	0.70
HYP	0.39	0.69	0.27

**Table 5:** Model evaluation of the LUDB dataset.

Method	AUC	Accuracy	F1	Precision	Recall	Params	Memory
ResNet34_1d [43]	0.870	0.841	0.552	0.520	0.588	16.604M	0.37 GB
ResNet34_2d [43]	0.863	0.850	0.578	0.543	0.619	21.281M	0.44 GB
DNN_zhu [44]	0.817	0.818	0.563	0.469	0.704	3.682M	0.12 GB
DSE-ResNet [10]	0.879	0.840	0.623	0.513	0.792	3.404M	0.33 GB
SE-ResNet1 [45]	0.907	0.897	0.697	0.686	0.708	58.147M	1.18 GB
SE-ResNet12 [45]	0.919	0.903	0.728	0.728	0.727	58.147M	1.18 GB
LightX3ECG [46]	0.857	0.898	0.711	0.673	0.753	5.583M	0.12 GB
MS-LTCAF (Ours)	0.942	0.920	0.745	0.794	0.701	24.585M	0.16 GB

**Table 6:** The experimental results of Lead-Temporal Co-Attention Mechanism.

Model	AUC	Accuracy	F1	Precision	Recall
W/O Lead Attention ^1^	0.877	0.858	0.699	0.773	0.638
W/O Temporal Attention ^2^	0.897	0.863	0.717	0.771	0.670
W/O Lead-Temporal Co-Attention ^3^	0.912	0.865	0.708	0.804	0.632
MS-LTCAF (Ours)	0.927	0.877	0.745	0.806	0.692

^1^ Using only temporal attention in the ablation experiment of Lead-Temporal Co-Attention Mechanism. ^2^ Using only lead attention in the ablation experiment of Lead-Temporal Co-Attention Mechanism. ^3^ Not using attention in the ablation experiment of Lead-Temporal Co-Attention Mechanism.

## Data Availability

The original contributions presented in the study are included in the article, further inquiries can be directed to the corresponding author.
